# Urban-rural difference in the lagged effects of PM2.5 and PM10 on COPD mortality in Chongqing, China

**DOI:** 10.1186/s12889-023-16113-9

**Published:** 2023-06-30

**Authors:** Aiping Gou, Guanzheng Tan, Xianbin Ding, Jiangbo Wang, Xiaoyan Lv, Chunyan Gou, Qiang Tan

**Affiliations:** 1 College of Ecological Technology and Engineering, Shanghai Institute of Technology, Shanghai, 201418 China; 2 Institute of Chronic and Non-communicable Disease Control and Prevention, Chongqing Center for Disease Control and Prevention, Chongqing, 400042 China; 3 College of Architecture, Nanjing Tech University, Nanjing, 211816 China; 4 Department of Acupuncture, Chongqing Traditional Chinese Medicine Hospital, Chongqing, 400021 China

**Keywords:** DLNMs, COPD, Urban-rural differences, PM2.5, PM10, Lagged effects

## Abstract

**Background:**

It is true that Chronic obstructive pulmonary disease (COPD) will increase social burden, especially in developing countries. Urban-rural differences in the lagged effects of PM2.5 and PM10 on COPD mortality remain unclear, in Chongqing, China.

**Methods:**

In this study, a distributed lag non-linear model (DLNMs) was established to describe the urban-rural differences in the lagged effects of PM2.5, PM10 and COPD mortality in Chongqing, using 312,917 deaths between 2015 and 2020.

**Results:**

According to the DLNMs results, COPD mortality in Chongqing increases with increasing PM2.5 and PM10 concentrations, and the relative risk (RR) of the overall 7-day cumulative effect is higher in rural areas than in urban areas. High values of RR in urban areas occurred at the beginning of exposure (Lag 0 ~ Lag 1). High values of RR in rural areas occur mainly during Lag 1 to Lag 2 and Lag 6 to Lag 7.

**Conclusion:**

Exposure to PM2.5 and PM10 is associated with an increased risk of COPD mortality in Chongqing, China. COPD mortality in urban areas has a high risk of increase in the initial phase of PM2.5 and PM10 exposure. There is a stronger lagging effect at high concentrations of PM2.5 and PM10 exposure in rural areas, which may further exacerbate inequalities in levels of health and urbanization.

## Introduction

Chronic obstructive pulmonary disease (COPD) is the leading cause of increased chronic morbidity, mortality, and medical cost worldwide [1], while acute exacerbations of COPD as acute events increase the socioeconomic burden [2]. It is particularly true in developing countries where the burden of COPD is greater than in developed countries. The prevalence of COPD in most regions of China is higher than that estimated model by the World Health Organization [3]. In 2013, the number of COPD-related deaths in China was 910,809, accounting for 31.1% of all such deaths in the world [4]. The exploration of COPD risk factors is important for effective public health policy making on COPD, which is an incomplete reversible airway obstruction disease characterized by airflow obstruction and chronic inflammation of the lungs and associated with an abnormal inflammatory response to harmful particles and gases in the lungs [5].

Particle matter (PM) is a combination of particles of different sizes and chemical properties that vary in time and space. Exposure to PM may lead to oxidative stress in cells and tissues, which can cause damage to cells [6]. Currently, there are extensive studies demonstrating that exposure to high concentrations of airborne pollutants (PM2.5, PM10) exacerbates COPD symptoms [7, 8], and increases the risk of COPD severity and death [9, 10]. These studies have mainly focused on urban areas [11, 12], and fewer on rural areas and regional differences [13]. Taking the administrative division of China as an example, if a prefecture-level city is taken as the study object, the urban-rural differences may be overlooked because a prefecture-level city unit contains rural areas in addition to urban built-up areas, and also there are differences in the spatial distribution of air particulate pollutants. Therefore, by exploring the urban-rural differences in air particulate pollutants and COPD mortality risk, we can provide effective decision support, which is important for the development of upstream planning and targeted interventions. The characteristics of urbanization in developing countries make urban-rural disparities more pronounced. In addition, the level of health risk management in developing countries lags behind that in developed countries in most cases [14, 15]. Therefore, the exploration of urban-rural differences in air particle pollutants and COPD mortality risk is particularly important for developing countries.

Air pollution increases the risk of all-cause mortality, cardiovascular mortality, and respiratory mortality. A study in China found a higher risk of air pollution-related mortality in rural areas, with rural residents being more sensitive to air pollution than urban residents [16]. Meanwhile, cancer mortality was higher in rural areas than in urban areas with long-term exposure to PM2.5 [17]. However, a study in the UK found that the burden of air pollution-related deaths was lowest in the poorest decile in rural areas [18]. Similarly, a study in California found lower mortality in urban areas compared to rural areas under chronic PM2.5 exposure [19]. The results on urban-rural differences in air pollution and mortality vary considerably across regions or countries.

Existing studies mainly rely on the daily average concentrations of atmospheric monitoring stations to detect the exposure-response relationship between air particle pollutants and COPD. The spatial distribution of air pollution particles is heterogeneous [20], and the spatial heterogeneity will be ignored if only the average daily concentrations at one or more stations represent the extent of air particle pollution over the entire region, which can lead to errors in the response of study results to regional differences. Therefore, we use raster data that can reflect the spatial heterogeneity and significant differences of airborne pollutants to respond to the changes in the airborne pollutant-COPD mortality relationship between urban and rural areas in developing countries in this paper. The potential reasons contributing to the formation of urban-rural differences are discussed, with the expectation of providing decision support for the development of health interventions and urban-rural planning policies in Chongqing, as well as for the health management of COPD in other developing countries.

## Data & methods

### Study area

**Fig. 1:**
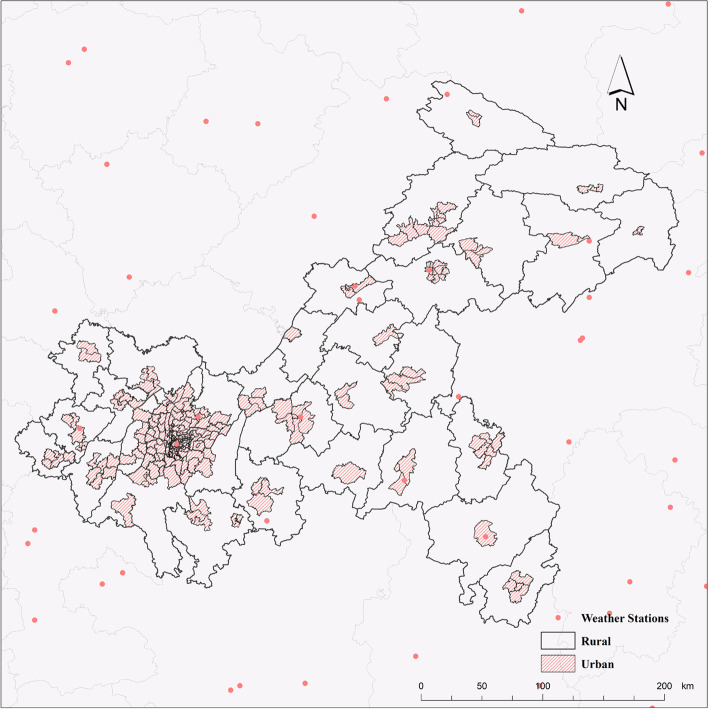
Urban and rural areas of Chongqing

The research area of this paper is Chongqing, which is located in the southwest of China inland and the upper reaches of the Yangtze River. It covers an area of 82,400 square kilometers and administers 38 districts and counties, including 26 districts, 8 counties and 4 autonomous counties. According to the sixth population census of China, the resident population of Chongqing is 28.84 million (2010) [21]. The urban-rural division in this study takes the administrative boundary of township and street level as the division unit. (Fig. 1).

### Data source

#### PM2.5, PM10 and population data

**Fig. 2:**
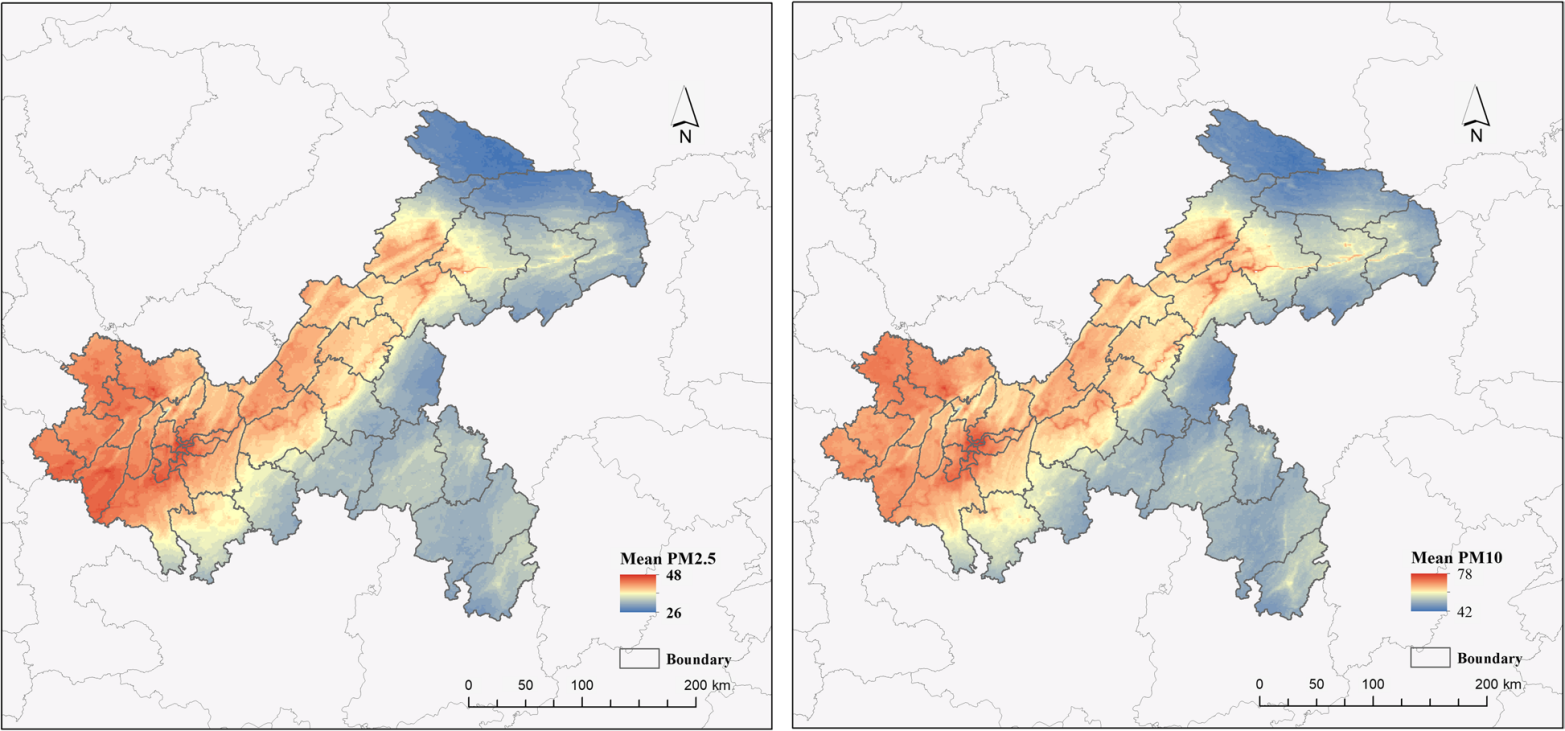
Left: Spatial distribution of average PM2.5 values in Chongqing from 2015 to 2020. Right: Spatial distribution of average PM10 values in Chongqing from 2015 to 2020

The air particle pollutants used in this paper are PM2.5 (ChinaHighPM2.5: Big Data Seamless 1 km Ground-level PM2.5 Dataset for China) and PM10 data (ChinaHighPM10: Big Data Seamless 1 km Ground-level PM10 Dataset for China), derived from the State Key Laboratory of Remote Sensing Science, Beijing Normal University, China [22, 23]. The dataset utilizes long-term high spatial resolution aerosol optical depths generated by the Moderate Resolution Imaging Spectroradiometer (MODIS) with Multi-Angle Atmospheric Correction (MAIAC) algorithm, and then a spatial-temporal tree (STET) model is used to estimate the daily grid data (2015–2020) of PM2.5 and PM10 concentrations with a high resolution of 1 km. The model can capture well variations in PM2.5 concentrations at different spatiotemporal scales, with higher accuracies (i.e., cross-validation coefficient of determination, CV-R^2^ = 0.86–0.90) and the PM10 product has an out-of-sample (out-of-station) cross-validation coefficient of determination (CV-R^2^) of 0.86 (0.82) [22, 23]. In this study, the raster data of Chongqing city were extracted by the Raster package in R language and divided into two regions, urban and rural, and the daily average concentrations of PM2.5 and PM10 in the two regions were calculated respectively. The spatial distribution of the daily average values of PM2.5 and PM10 in Chongqing during 2015–2020 shows that the high values are mainly concentrated in the western and central-northern regions of Chongqing, which coincides with the distribution of the western urban agglomeration. In addition, the daily average concentration of PM10 is higher than that of PM2.5 (Fig. 2).

The population data were obtained from the sixth census data (2010) published by the Chongqing Bureau of Statistics [24], and the population of the urban area was 14.5 million and rural area was 14.34 million after partitioning by Fig. 1.

#### COPD mortality data

The data on COPD deaths in this paper were obtained from 312917 day-by-day death data (2015-2020) of all districts and counties collected by the Chongqing Center for Disease Control and Prevention. The dataset was geocoded by Python, and then the coded data were divided into two-time series datasets for urban and rural areas.

#### Temperature and Relative humidity data

As the confounding factor for constructing the DLNMs model, they were obtained from the dataset V3.0 of Chinese terrestrial climate information from the China Meteorological Data Center [25], which was interpolated by inverse distance weights. Finally, the daily average temperature value and relative humidity of Chongqing City from 2015 to 2020 are formed.

### Statistic analysis

The analysis process in this paper will be divided into two stages. In the first stage, a regression model is chosen to analyze the exposure-response relationship. The second stage performs hypothesis testing on the results of the two regions to find out whether the difference is due to sampling error.

Air pollution, meteorological elements and other exposure factors have a degree of persistence and lag in health effects [26–28]. That is, when the population is exposed to air pollution, their health indicator status is not only related to the degree of exposure on that day, but also may be the result of the combined effects of the previous exposure [29]. Therefore, the delayed effects should be taken into account to assess the exposure-response relationship between PM2.5, PM10, and COPD mortality. The Pearson correlation coefficient test [30] for PM2.5, PM10, and COPD mortality found a potential nonlinear relationship between them. The lagged effects are generally described by distributed lag models (DLMs), which were originally used in economics studies [31] and later introduced in the quantitative assessment of environmental factors and health effects [32, 33]. Traditional DLMs assume a linear relationship between exposure and effects of environmental factors as a premise, but it is often not linear in reality [34] with many potential nonlinear factors. In order to describe both the nonlinear relationship and the lagged effects between exposure and outcome, new model frameworks DLNMs are proposed [35].

Therefore, the DLNMs model, based on the ideological basis of generalized linear model and generalized summation model, is used in the first phase of this study. It effectively assesses exposure-response relationships and lagged effects in time series data by incorporating cross-basis functions. the basic expression of the DLNM model is:

1 $$g\left(\mu_t\right)=\alpha+\sum_{j=1}^js_j\left(x_{tj};\beta_j\right)+\sum_{k=1}^ky_ku_{tk}$$

Where ${\mu }_{t}=\text{E}\left({Y}_{t}\right)$, ${Y}_{t}$ represents the outcome variable, which fits multiple probability distributions, and $\text{E}\left({Y}_{t}\right)$ represents the expectation of the dependent variable *Y* at time *t*;$g$ represents the connection function; ${s}_{j}$ represents the functional relationship between ${x}_{j}$ and $\text{E}\left({Y}_{t}\right)$, and ${u}_{k}$ represents other variables that have a linear relationship with $\text{E}\left({Y}_{t}\right)$; $\beta , \gamma$ represent the parameter vectors of ${x}_{j},{u}_{k}$, respectively. ${s}_{j}$ represents the basis function of the independent variable ${x}_{j}$, by choosing different basis functions, ${x}_{j}$ can be converted into a new set of variables to be included in the design matrix of the model and parameter estimation. The common basis functions include polynomial functions, threshold functions and spline functions, among which natural cubic spline is more widely chosen, the distribution of dependent variables can be better described by the transformation of independent variables into proper basis functions.

In this study, the natural cubic strip (type="ns”) was used to control the exposure-response relationship between airborne pollution and COPD mortality in the cross-sectional base setting. Considering that there is no absolute safe level of PM2.5 and PM10, the reference value is set to 0. B-spline (type="bs”) is used to control the lag effect with a degree of freedom (df) of 4. The maximum lag length is 7. The generalized linear model (GLM) with standard quasi-Poisson as the link function is chosen as the regression model, which contains covariates such as the numbers of series of natural cubic splines with degree of freedom 4/year controlling for long-term trends, dummy variables controlling for day-of-week effects, and the day-by-day average temperature in Chongqing as a confounding factor. The specific expressions are as follows:

2 $$g\left(deaths\right)=b_0+b_1\ast cb.\;PM+dow+c\left(Rh\right)+c\left(Temp\right)+ns\left(Time\right)$$

Where $g\left(\right)$ represents the connection function; $Deaths$ represents the expectation of the COPD mortality; $cb.PM$ denotes the cross-basis of PM2.5 or PM10; $dow$ is a week dummy variable; $c\left(Rh\right)$ and $c\left( Temp \right)$ are the average daily relative humidity and average daily temperature respectively; $ns\left( Time \right)$ is the number of time series; ${b}_{0}$ and ${b}_{1}$are the intercepts and regression coefficients.

In the second stage, the urban-rural differences in the interrelationship between airborne particulate pollutants and COPD mortality were hypothesized to investigate whether the differences were due to sample error. In order to suggest the robustness of the results, a sensitivity analysis of the model is carried out. In this study, sensitivity analysis of the model is carried out by changing the model lag days from 7 to 15 d and controlling for the relative humidity variable.

The statistical analysis and spatial data processing in this paper are based on the R software (R version 4.2.0) “dlnm” [35], “splines” (R Core Team, 2022), the “raster” package [36], and Arcgis 10.5.

## Result

### Descriptive statistics

**Fig. 3:**
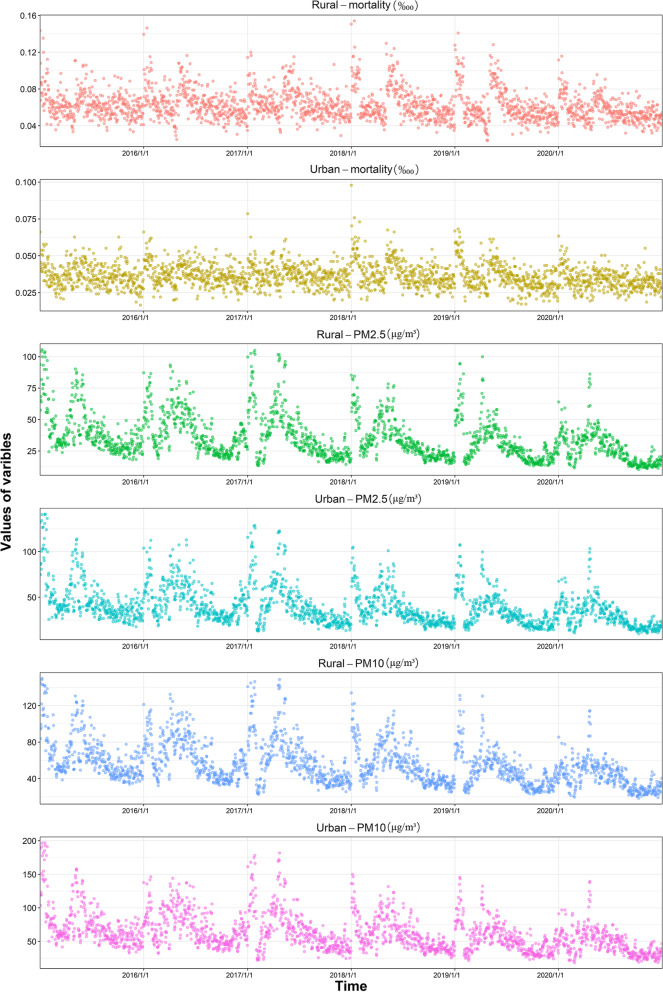
Temporal trends in variables from 2015 to 2020

The daily mean, standard deviation and maximum values of COPD mortality rate per 10,000 people, PM2.5 and PM10 in urban and rural areas of Chongqing from 2015 to 2020 are shown in Table 1. The COPD mortality rate per 10,000 in rural areas is 1.75 times that in urban areas, but the mean values of PM2.5 and PM10 are 0.90 times and 0.89 times that in urban areas, respectively. As shown in Fig. 3, the temporal variation of PM2.5 and PM10 has a certain periodicity, with high values mainly in winter and summer, and the high COPD mortality rate per 10,000 is also mainly in winter and summer.

**Table 1:** Descriptive statistics of main data

	Rural	Urban
COPD Mortality (‱)		
Min	0.024	0.017
Max	0.154	0.098
Median	0.059	0.035
Mean(day)	0.063	0.036
SD	0.0169	0.0084
PM2.5 (µg/m³)		
Min	10.34	9.94
Max	105.58	141.17
Median	31.11	34.42
Mean(day)	35.27	39.83
SD	17.43	21.15
PM10 (µg/m³)		
Min	18.48	18.42
Max	149.9	196.96
Median	51.11	56.92
Mean(day)	55.99	62.59
SD	23.46	28.06

*Min *Minimum value from 2015 to 2020, *Max *Maximum value from 2015 to 2020, *Median *Daily average from 2015 to 2020, *SD *Standard deviation

### Pearson correlation coefficient

**Fig. 4:**
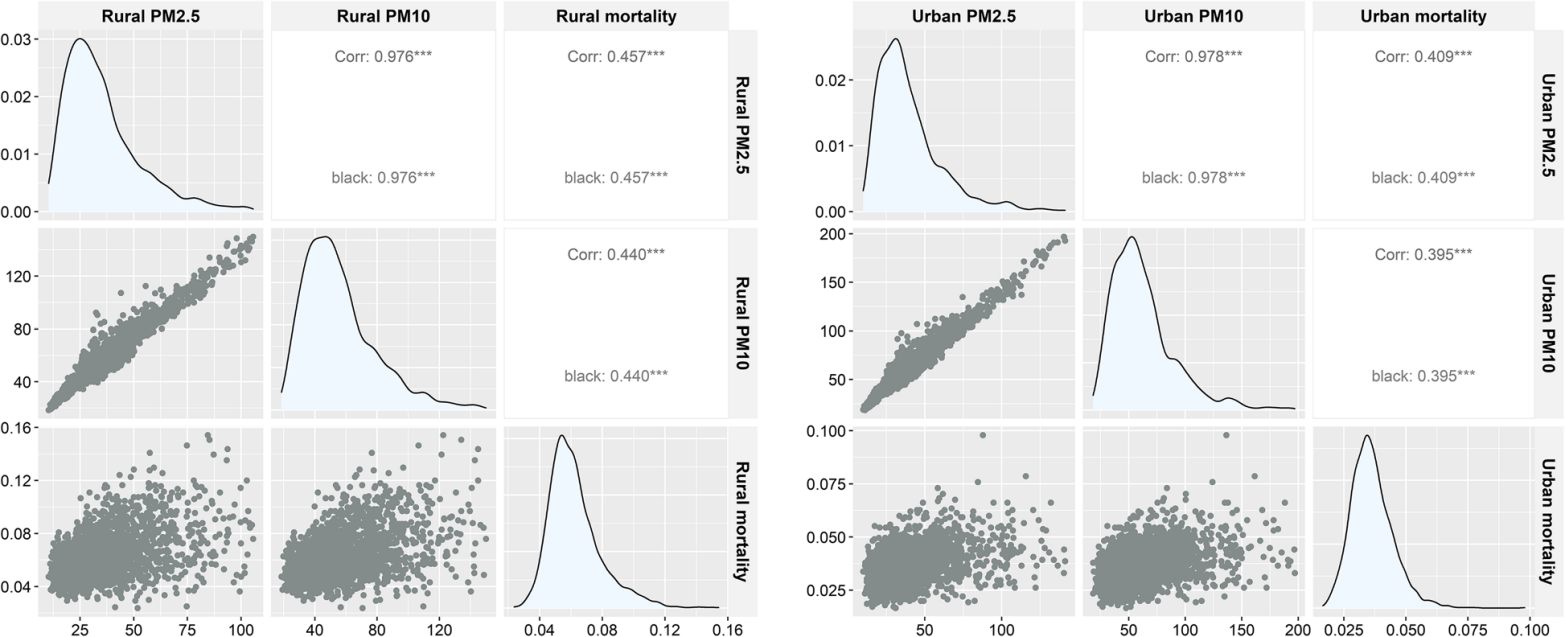
Pearson correlation analysis. There was a significant linear correlation between urban and rural PM2.5 and PM10 (*P* < 0.01). There was a significant correlation between PM2.5, PM10 and COPD mortality (*P* < 0.01)

Pearson correlation analyses were performed between PM2.5, PM10 and COPD mortality in urban and rural areas of Chongqing, respectively (Fig. 4). The correlation coefficients between PM2.5, PM10 and COPD mortality in urban areas were 0.409 (*P*-value < 0.001) and 0.395 (*P*-value < 0.001), respectively, with a significant moderate positive correlation; the correlation coefficient between PM2.5 and PM10 in urban areas was 0.978 (*P*-value < 0.001), showing significant high intensity correlation. The correlation coefficients between PM2.5, PM10 and COPD mortality in rural areas were 0.457 (*P*-value < 0.001) and 0.440 (*P*-value < 0.001), respectively, with a significant moderate positive correlation and a higher correlation coefficient comparing to that in urban areas. Similar to urban areas, rural areas showed a significant high intensity correlation between PM2.5 and PM10 with a correlation coefficient of 0.976 (*P*-value < 0.001).

### Lagged effects of PM2.5 on COPD mortality and urban-rural differences

**Fig. 5:**
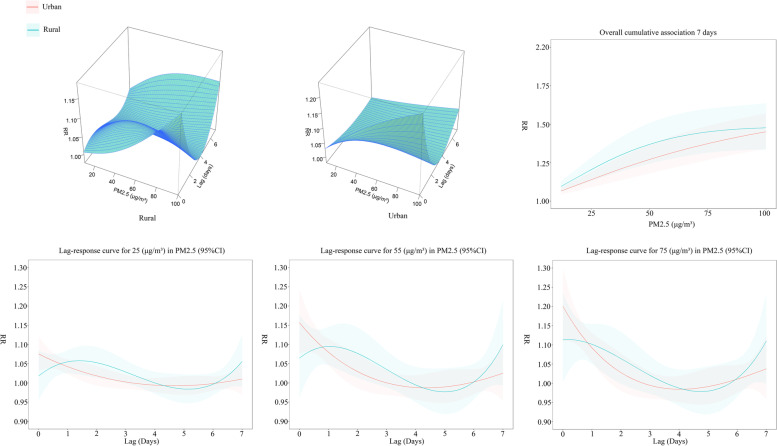
Lagged effect of PM2.5 on COPD mortality. Three-dimensional plots depict the overall exposure-response relationship between PM2.5 and COPD mortality. The overall 7-day cumulative effect responds to the change in RR values with increasing PM2.5 concentrations at a 7-day lag time, where the shaded areas are 95% confidence intervals, with blue representing rural areas and red representing urban areas. The Lag-response curves in the lower half depict the variation of RR values with increasing lag time for PM2.5 at concentrations of 25 µg/m³, 55 µg/m³, and 75 µg/m³

Figure 5 shows the lagged effect of PM2.5 on COPD mortality in Chongqing and the urban-rural differences. In general, the PM2.5 effect on COPD mortality was non-linear, with the main trend of the PM2.5 effect on COPD mortality decreasing and then increasing with increasing lag time in both urban and rural areas.

Three-dimensional plots depict the overall exposure-response relationship between PM2.5 and COPD mortality. At high concentrations, the effect of PM2.5 on COPD mortality is particularly pronounced in urban versus rural areas at Lag 0–1 and Lag 6–7. According to the Lag 7-day accumulation lag effect (reference value: 0 µg/m³), the RR values increase with increasing PM2.5 concentrations and are higher in rural areas than in urban areas at all PM2.5 concentrations. The 7-day accumulation RR values for PM2.5 in urban areas at 25 µg/m³, 55 µg/m³ and 75 µg/m³ concentrations were 1.139 (95% CI: 1.089 ~ 1.191), 1.294 (95% CI: 1.198 ~ 1.397), and 1.374 (95% CI: 1.267 ~ 1.490). The 7-day cumulative RR values of PM2.5 in rural areas were 1.204 (95%CI: 1.130 ~ 1.283), 1.391 (95%CI: 1.261 ~ 1.535), and 1.450 (95%CI: 1.314 ~ 1.600) at concentrations of 25 µg/m³, 55 µg/m³ and 75 µg/m³, respectively. The urban-rural gap in RR values increases and then decreases as the concentration of PM2.5 increases.

In addition, Fig. 5 shows the change in the lag effect compared to the reference value (0 µg/m³) for 7 days plotted at 25 µg/m³, 55 µg/m³ and 75 µg/m³ concentrations to reflect the change in the lag effect at low, medium and high concentrations of PM2.5. High RR values in urban areas occur at the beginning of exposure (Lag 0 ~ Lag 1) and gradually decrease with increasing lag time during Lag0-Lag 4. High RR values in rural areas occur during Lag 1 to Lag 2 and Lag 6 to Lag 7, but with increasing PM2.5 concentrations high RR values occur during Lag 0 to Lag 1 and Lag 6 to Lag 7. In the early exposure period (Lag 0 ~ Lag 1), urban areas had higher RR values than rural areas, in the lag1 ~ lag4 period rural areas had higher RR values than urban areas, and in the late lag period (Lag 6 ~ Lag 7), rural areas had higher RR values than urban areas again.

### Lagged effects of PM10 on COPD mortality and urban-rural differences

**Fig. 6:**
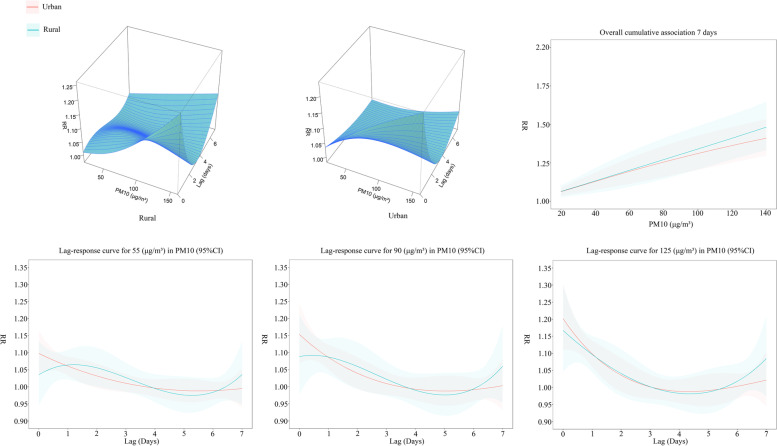
Lagged effect of PM10 on COPD mortality. Three-dimensional plots depict the overall exposure-response relationship between PM10 and COPD mortality. The overall 7-day cumulative effect responds to the change in RR values with increasing PM10 concentrations at a 7-day lag time, where the shaded areas are 95% confidence intervals, with blue representing rural areas and red representing urban areas. The Lag-response curves in the lower half depict the variation of RR values with increasing lag time for PM10 at concentrations of 55 µg/m³, 90 µg/m³, and 125 µg/m³

Figure 6 shows the lagged effect of PM10 on COPD mortality in Chongqing and the urban-rural differences. Three-dimensional plots depict the overall exposure-response relationship between PM2.5 and COPD mortality. At high concentrations, the effect of PM2.5 on COPD mortality is particularly pronounced in urban versus rural areas at Lag 0–1 and Lag 6–7.

According to the Lag 7-day accumulation lag effect (reference value: 0 µg/m³), the RR values increase with increasing PM10 concentrations and are higher in rural areas than in urban areas at all PM10 concentrations. The 7-day accumulation RR values for PM10 in urban areas at 55 µg/m³, 90 µg/m³ and 125 µg/m³ concentrations were 1.139 (95% CI: 1.089 ~ 1.191), 1.294 (95% CI: 1.198 ~ 1.397), and 1.374 (95% CI: 1.267 ~ 1.490). The 7-day cumulative RR values of PM2.5 in rural areas were (95%CI: 1.130 ~ 1.283), 1.391 (95%CI: 1.261 ~ 1.535), and 1.450 (95%CI: 1.314 ~ 1.600) at concentrations of 55 µg/m³, 90 µg/m³, and 125 µg/m³, respectively. The urban-rural gap in RR values increases and then decreases as the concentration of PM2.5 increases.

Figure 6 also shows the change in lag effect compared to the reference value (0 µg/m³) for 7 days plotted at 55 µg/m³, 90 µg/m³ and 125 µg/m³ concentrations to reflect the change in lag effect at low, medium and high concentrations of PM10. High RR values in urban areas occur at the beginning of exposure (Lag 0 ~ Lag 1) and gradually decrease with increasing lag time during Lag 0 to Lag 4. High RR values in rural areas occur during Lag 1 to Lag 2 and Lag 6 to Lag 7, but with increasing PM2.5 concentrations high RR values occur during Lag 0 to Lag 1 and Lag 6 to Lag 7. In the early exposure period (Lag 0 ~ Lag 1), urban areas had higher RR values than rural areas, in the Lag 1 to Lag 4-period rural areas had higher RR values than urban areas, and in the late lag period (Lag 6 ~ Lag 7), rural areas had higher RR values than urban areas again.

### Hypothesis testing

This section investigates whether the urban-rural differences in the interrelationship between airborne particulate pollutants and COPD mortality are due to sample error by conducting a hypothesis test on the results of the urban-rural differences in the correlation between PM2.5, PM10 and RR values. Firstly, Shapiro-Wilk test was performed on the outcome data of PM2.5, PM10 on the lagged effect of COPD mortality, and PM2.5 results in urban and rural areas did not conform to normal distribution after Shapiro-Wilk test (*P*-value < 0.01). Therefore, under the Wilcoxon’s rank sum test, there was a statistically significant difference between the results of the lagged effect of PM2.5 on COPD mortality in urban and rural areas (*P*-value < 0.01). The PM10 results for urban and rural areas also did not conform to a normal distribution after the Shapiro-Wilk test (*P*-value < 0.01). Therefore, the same Wilcoxon’s rank sum test was used and a statistically significant difference was found between the results of the lagged effect of PM10 on COPD mortality in urban and rural areas (*P*-value < 0.01).

### Sensitivity analysis

**Fig. 7:**
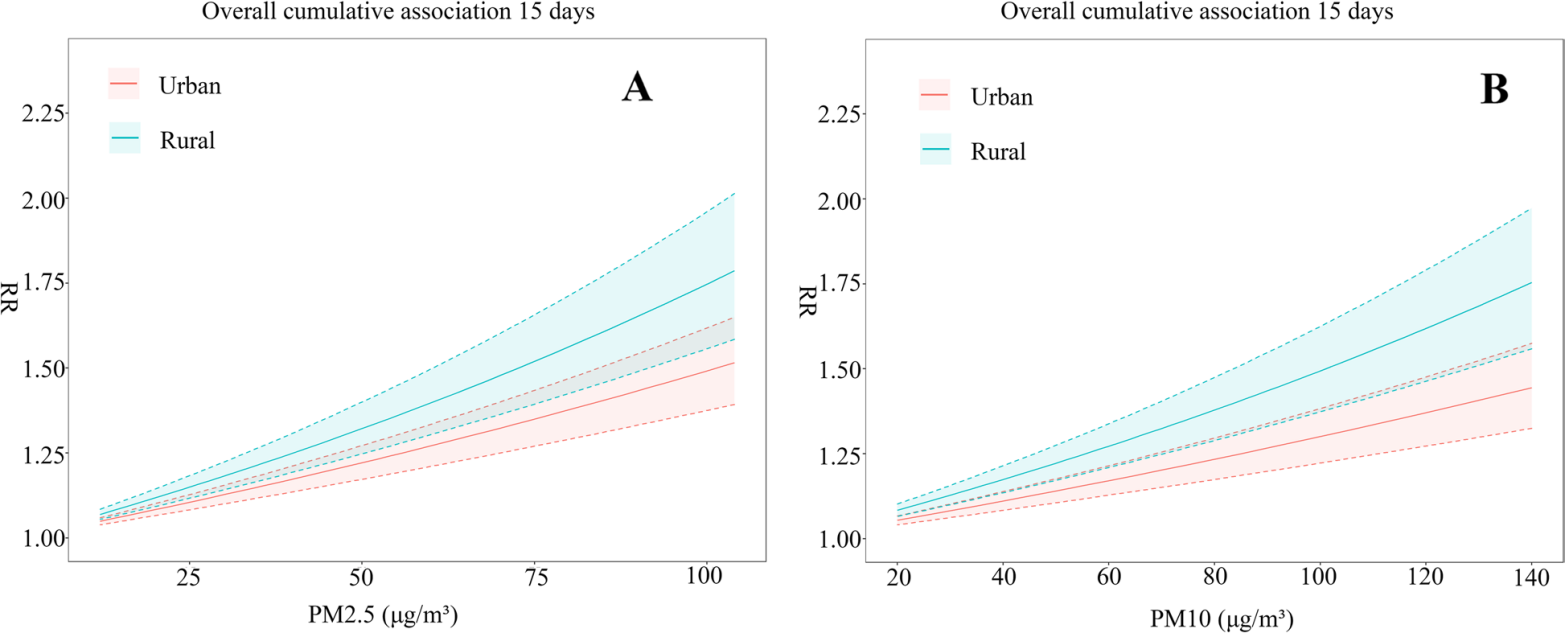
A: Cumulative 15-day lag effect of different concentrations of PM2.5; B: Cumulative 15-day lag effect of different concentrations of PM10. The shaded areas are 95% confidence intervals, with blue representing rural areas and red representing urban areas

Sensitivity analysis is carried out by varying the maximum lag time of the model, the degrees of freedom of the variables and controlling the relative humidity variable. Similar results can be seen in Fig. 7, where RR values increase with increasing PM2.5 and PM10 concentrations, with higher RR values at each PM2.5 and PM10 concentration in rural areas than in urban areas.

## Discussion and limitations

Exposure to PM2.5, PM10 was found to be positively associated with the risk of COPD mortality, similar to previous studies, after a Meta-analysis of the large literature by DeVries et al. [13] who concluded that PM2.5 was positively correlated with mortality of COPD. This study also found that the cumulative lagged effects of PM2.5 and PM10 on the risk of COPD mortality in the same region showed roughly the same trend, first decreasing and then increasing with the lag length, but there were some differences between urban and rural areas.

According to the model results of the DLNMs, the RR of the overall 7-day cumulative effect of PM2.5 and PM10 is higher in rural areas than in urban areas. The RR in rural areas is significantly higher than that in urban areas at the later stages of exposure as the lag time changed. This may imply that there is still a gap between medical services, economic level and awareness of residents in the rural areas and the urban areas of Chongqing, which means residents who are in some backward rural areas may not have timely access to high levels of medical services and awareness of air pollution protection when exposed to air pollution. The relative risk tends to be higher in urban areas than in rural areas at the beginning of exposure, probably due to the prevalence of cars and high-density built environment, urban residents are more likely to be exposed to PM2.5 and PM10, but the relative risk decreases gradually with increasing lag time due to better medical care.

The strongest effect of PM2.5 on COPD mortality risk in urban versus rural areas occurred at Lag 0–1, similar to the results of a study by Wu et al. [12] in Beijing. A study by Hueglin et al. [37] in Switzerland found that the pollution levels of PM2.5 and PM10 decreased gradually along the urban phase, suburban, and rural areas; and a study by Jiang et al. [38] on PM2.5 concentrations in 11 cities in China found that PM2.5 emission rates were higher in urban areas than in rural areas. However, the RR of 7-day cumulative COPD mortality at high PM2.5 and PM10 exposures was higher in rural areas of Chongqing than in urban areas in this study. From the perspective of overall regional characteristics, this may be due to the fact that there are fewer health services available in rural areas than in urban areas and the possibility of unequal distribution of health resources. Meanwhile, the quality of housing in rural areas is not sufficient to mitigate air pollution exposure [39], especially in developing countries. In terms of population characteristics, due to the difference of lifestyle between urban and rural residents, rural residents have more outdoor activities, resulting in higher outdoor air pollution exposure than urban areas [40]. In addition, income level, occupational type, and educational background all influence their air pollution susceptibility; For example, low-income people face higher vulnerability to air pollution [41, 42], whereas most blue-collar workers and people with low education levels have lower incomes [39, 43], and people with low education levels may also lack knowledge about air pollution disease prevention [44].

This study revealed the urban-rural differences between PM2.5, PM10 and COPD risk of death in developing countries and their lagging effects. The PM2.5 and PM10 data were collected using a high-precision day-by-day raster dataset, which can better reflect the spatial heterogeneity of PM2.5 and PM10 than the mean values of regional stations; secondly, the urban-rural differences between PM2.5, PM10 exposure and COPD mortality were revealed by geocoding and DLNM models, which provide a basis for health management and COPD prevention policy formulation at the street and township level in Chongqing, China, and also provides a reference and experience for other developing countries.

However, there are some limitations in this study at the same time. First, there is no unified definition and standard for the division of urban and rural areas in China, and this study distinguishes urban and rural areas at the scale of existing administrative units at the township level by referring to satellite image maps, so it is not completely accurate to distinguish urban and rural areas; and some cases is not accurately obtained due to incomplete addresses; Secondly, the absence of long-term day-by-day data and fewer confounders may lead to errors in the prediction results; finally, this is a regional study of urban-rural differences between PM2.5, PM10 exposure and COPD mortality interrelationships, but it does not involve the exposure of individual cases, which may bias its true estimation.

## Conclusion

Exposure to PM2.5 and PM10 is associated with an increased risk of COPD mortality in Chongqing, China. COPD mortality in urban areas has a high risk of increase in the initial phase of PM2.5 and PM10 exposure, therefore the government should strengthen the control of PM2.5 and PM10 emissions. There is a stronger lagging effect at high concentrations of PM2.5 and PM10 exposure in rural areas, which may further exacerbate inequalities in levels of health and urbanization. Therefore, more attention needs to be given to rural areas in the development of COPD health risk management policies and the allocation of health care resources. For individuals with COPD, it is also important to reduce outdoor activities and take protective measures during periods of high PM2.5 and PM10 concentrations.

## Data Availability

The data of PM2.5 and PM10 can get from [ChinaHighPM2.5: Big Data Seamless 1 km Ground-level PM2.5 Dataset for China] at [10.5281/zenodo.6398971] and [ChinaHighPM10: Big Data Seamless 1 km Ground-level PM10 Dataset for China] at [10.5281/zenodo.6449937]. The population data can get from [Chongqing Bureau of Statistics] at [http://tjj.cq.gov.cn/wap.html]. Climate data obtained from the dataset V3.0 of Chinese terrestrial climate information from the China Meteorological Data Center [https://data.cma.cn/]. But the COPD mortality data will be made available from the corresponding author on reasonable request due to privacy and ethical restrictions.
